# More Adventures in Photodynamic Therapy

**DOI:** 10.3390/ijms160715188

**Published:** 2015-07-03

**Authors:** David Kessel

**Affiliations:** Department of Pharmacology, Wayne State University School of Medicine, 540 E Canfield Street, Detroit, MI 48201, USA; E-Mail: dhkessel@med.wayne.edu; Tel.: +1-313-577-1787

**Keywords:** apoptosis, autophagy, photodynamic therapy

## Abstract

Photodynamic therapy is a procedure that can provide a selective eradication of neoplastic disease if sufficient drug, light, and oxygen are available. As this description suggests, it involves the photosensitization of malignant tissues to irradiation with photons in the visible range. While not suitable for tumors at unknown loci, it can be of use for eradication of cancer at surgical margins and therapy at sites where substantial surgery might otherwise be involved. Drug development has been delayed by several factors including the reluctance of major pharmaceutical firms in the United States to invest in this technology along with some unwise approaches in the past.

## 1. Introduction

This commentary will discuss the background and potential utility of photodynamic therapy in the treatment of cancer, with a brief mention of other implications. Some thoughts on future directions or lack thereof are also included.

## 2. Discussion

While the origin of photodynamic effects can be traced back to the early Egyptians who used sunlight to bleach fabrics, for most of us, it began with work by Raab and von Tapiener in the early 20th century. Raab observed that light could kill a microbial population photosensitized with an acridine dye. A variety of further explorations followed, with a low level of research going on in ensuing years. This included a report at the first meeting of the American Society for Photobiology. An incandescent light bulb was used as the light source. It seems safe to propose that the modern era began with a series of investigations by the Roswell Park group led by Thomas Dougherty in the 1970s [1]. There was also a group of “cutting edge” physicians and research personnel who discovered how a relatively low light dose could selectively control cancers that were untouched or minimally affected by conventional chemotherapy or ionizing radiation. This phase of drug development, along with more recent discoveries, has been amply reviewed [2,3,4].

A critical precursor to Dougherty’s work was Dr. Sam Schwartz, at the Mayo clinic, who had an interest in porphyrias and had experimented with a variety of porphyrins that appeared to localize in neoplastic tissues. Schwartz had attempted to purify hematoporphyrin, one of the porphyrins that is obtained in high yield when the protoporphyrin in hemoglobin is treated with strong acids. Using a type of chemical logic that can prevail in the untrained, Schwartz treated hematoporphyrin with a mixture of sulfuric and acetic acids, followed by neutralization with NaOH. The rationale for this was never made clear, but the result was a very complex mixture, found to be a superior tumor-localizing product. Schwartz named this material HPD (hematoporphyrin derivative), having no clear information on what he had created. Considerable effort was devoted to working out exactly what was in there. The definitive studies were done by Pandey who reported that a collection of porphyrin ethers were the major useful ingredients [5,6].

Dougherty’s group had assumed that a product intended for use solely within the State of New York was exempt from the US Food and Drug Administration (FDA), but were eventually enlightened. The FDA eventually did approve a purified version of HPD (Photofrin) some anti-tumor indications. Early progress was sufficiently impressive to initiate a widespread research effort. Meanwhile a series of other investigators began creating single molecules that could also localize in tumors and eradicate them upon irradiation. Thus, in spite of some early fears, there was nothing “magic” about the complex mixture “HPD”. Dougherty and others worked out that both light and oxygen were necessary for the phototoxic effect. It was also discovered that an important element of the overall effect was also an element of vascular shutdown, inflammation and the evoking of immune responses.

My introduction to the field occurred in 1976 when I received a letter from the (National Institutes of Health) NIH asking me to examine a collection of brown powders which made up the porphyrin collection at the NIH. Later analysis revealed that each of these was actually a mixture of porphyrins of which at least one component corresponded to the structure provided. The early workers were anxious to compare notes, and there followed a series of conferences beginning with a 1977 meeting in Buffalo, organized by Dougherty. There was considerable interest in photodynamic therapy (PDT) in Japan where Drs. Hayata and Kato at the Tokyo Medical College were instrumental in introducing PDT into the clinical treatment of lung cancer. Later advances resulted in the design of light sources involving lasers and fiber optics; easier to manage than a 1000-watt xenon lamp with assorted filters designed to remove infra-red (heat), ultraviolet and other extraneous wavelengths of light. I had attempted to interest the Ciba Foundation (London) in a Symposium on the topic of PDT in the early 1980s, but this resulted in only a 1982 Discussion Group meeting. A Ciba Symposium on PDT was eventually held in London in 1989 with invited contributions and discussions provided by many leaders in PDT research [7]. It is unfortunate that Ciba (later absorbed by Novartis) has abandoned the symposium-sponsoring business, with the old Ciba House on Portland Street in London now a “for hire” conference center.

At that 1982 Discussion Group session, one of the participants was Prof. Alastair Currie, who had identified apoptosis as a common mode of cell death [8]. He asked if apoptosis might be involved in the mode of action of PDT. At the time, there was no persuasive hypothesis on PDT-induced cell death modes: some type of necrosis was generally assumed to be involved. As late as 1988, when I edited a two-volume work for CRC Press incorporating the latest thoughts on death mechanisms [9], there was only an appreciation that oxygen was required for the success of PDT and that there was cell damage wherever one looked. A few years later, explanations for the mechanism of PDT-induced direct cell death began to appear. Oleinick’s group identified apoptosis as a cell-death mode associated with photokilling [10]. This explained the ability of a relatively low light dose to evoke a substantial degree of cell death. Apoptosis is an irreversible pathway to cell death and can be triggered by release of cytochrome c molecules into the cytoplasm. The initial level of cell perturbation is therefore substantially less that what is required for direct tumor destruction, e.g., with CO_2_ or eximer lasers. We later reported that a major target of many photosensitizers is the anti-apoptotic protein termed Bcl-2 [11]. Loss of the functionality of this protein is a also known initiator of apoptosis.

A variety of mitigating factors then began to be uncovered that could promote or suppress cell death. The evaluation of the voluminous data on pathways of photokilling is difficult since many of the events characterized are common to any toxic phenomenon. As cells begin to die, an array of enzymes are activated or inactivated and many things begin to happen. There are estimates that the executioner caspases act on hundreds of proteins; dysfunctional mitochondria produce reactive oxygen species and a collection of survival responses appear. Much of this is not unique to PDT, and it is unclear whether interference with these responses can selectively promote or otherwise alter PDT efficacy.

A known influence on the effectiveness PDT is the process of autophagy whereby cells recycle organelles. This is mainly a response to starvation, with the autophagic process leading to formation of vesicles that engulf sub-cellular material. A review on the role of autophagy in the context of PDT has been published [12]. Fusion with lysosomes then leads to release of lysosomal proteases, a digestive process and release of basic biologic building blocks into the cytoplasm where these can be used to maintain cellular processes. While loss of autophagy can promote photokilling, in the absence of apoptosis, autophagy might become a death pathway [13]. A role for autophagy in cytoprotection is illustrated by the finding that “cancer stem cells” appear to be protected from PDT photokilling by an increased capacity for autophagy [14].

Autophagy can also play a role in enhanced immune recognition of neoplasia [15]. This may represent yet another route to improved cancer control by photodamage that is not elicited by conventional chemotherapy.

An interesting new development is the use of PDT as part of an anti-tumor combination. Hasan’s group has pioneered here and has also explored opportunities for use of nanotechnology to promote PDT selectivity [16,17,18]. A 2006 report discussed the markedly increased efficacy of PDT in an animal tumor model when low-dose lysosomal photodamage preceded what was intended to be PDT directed against the tumor vasculature [19]. While the effect was clearly demonstrable, vascular shut-down did not appear to be involved. We have recently explored this effect and have ascribed the effects to amplification of pro-apoptotic signals [20,21]. Whether this modality can play a role in clinical PDT remains to be established.

## 3. Conclusions

What is the future of PDT? At present, there are indications for which this procedure has been shown to be highly effective. A persisting problem after cancer surgery is the persistance of malignant cells at surgical margins [22]. Based on its ability to selectively eradicate such cells, PDT is becoming a useful therapy for treatment of head and neck cancer [23]. Another important indication for PDT was in the treatment of macular degeneration and this formed a highly profitable venture until anti-vascular endothelial growth factor therapy began to replace PDT [24].

The most successful use of clinical PDT appears to be in Asia and Europe where the barriers to widespread utilization are not so steep as in the United States. This is perhaps because the reimbursement procedures and opportunities for research support are different than in the United States. Clinical trials are very expensive here, and it has been estimated that the cost of developing a single drug can be in the hundreds of millions of dollars. This hurdle is a difficult one to overcome. While the idea of unleashing a collection of unproven protocols into clinical practice is clearly not acceptable, the high price of drug development means that the road is very precarious. The use of PDT for treatment of macular degeneration did represent a notable financial success. As interest by the pharmaceutical industry begins to wane, this is reflected in levels of support by the granting agencies. There are currently three major groups supported by NIH program project grants (Penn, Harvard/Massachusetts General Hospital/Roswell Park). Support for other research efforts is more limited, perhaps a function of the limited interest in the field by major pharmaceutical organizations.
